# Trends in Compressive Sensing for EEG Signal Processing Applications

**DOI:** 10.3390/s20133703

**Published:** 2020-07-02

**Authors:** Dharmendra Gurve, Denis Delisle-Rodriguez, Teodiano Bastos-Filho, Sridhar Krishnan

**Affiliations:** 1 Department of Electrical, Computer, and Biomedical Engineering, Ryerson University, Toronto, ON M5B 2K3, Canada; krishnan@ryerson.ca; 2 Postgraduate Program in Electrical Engineering, Federal University of Espirito Santo, Vitoria 29075-910, Brazil; delisle05@gmail.com (D.D.-R.); teodiano.bastos@ufes.br (T.B.-F.)

**Keywords:** compressive sensing, EEG, low power BCIs, neurofeedback, assistive technology, sampling, data acquisition

## Abstract

The tremendous progress of big data acquisition and processing in the field of neural engineering has enabled a better understanding of the patient’s brain disorders with their neural rehabilitation, restoration, detection, and diagnosis. An integration of compressive sensing (CS) and neural engineering emerges as a new research area, aiming to deal with a large volume of neurological data for fast speed, long-term, and energy-saving purposes. Furthermore, electroencephalography (EEG) signals for brain–computer interfaces (BCIs) have shown to be very promising, with diverse neuroscience applications. In this review, we focused on EEG-based approaches which have benefited from CS in achieving fast and energy-saving solutions. In particular, we examine the current practices, scientific opportunities, and challenges of CS in the growing field of BCIs. We emphasized on summarizing major CS reconstruction algorithms, the sparse basis, and the measurement matrix used in CS to process the EEG signal. This literature review suggests that the selection of a suitable reconstruction algorithm, sparse basis, and measurement matrix can help to improve the performance of current CS-based EEG studies. In this paper, we also aim at providing an overview of the reconstruction free CS approach and the related literature in the field. Finally, we discuss the opportunities and challenges that arise from pushing the integration of the CS framework for BCI applications.

## 1. Introduction

As the fourth industrial revolution has accelerated, big data is growing in the field of healthcare, and the field of neural engineering is no exception. The neurological big brain data comes from a number of sources including hospitals, research labs, and consumer-directed neurotechnological devices. This brain data may be used to understand the brain structure and function for improving an individual’s health. For instance, the Image and Data Archive (IDA), a neurocognitive data archiving, and sharing platform, presently contains over 130 terabytes of neurological data to study the brain structure and functions [1]. Since each wire implanted in the brain tracks the activity of multiple neurons at once, it is difficult to identify which electrical bursts belong to which cell because of the biological complexity of the brain. Neuroscientists are now studying how an entire living brain functions by understanding different neuronal dynamics. For instance, one of the initial researches towards a scalable high-bandwidth brain research and an integrated model proposed by Musk [2] can collect brain signals from 3072 electrodes simultaneously. Few initial studies on other species brains also confirm the immensity of cortical activity. For example, 20 min of neural activity in a mouse brain recording produces about 500 petabytes of flickering data [3]. In another brain study, the brain needed a few terabytes of images to reconstruct 1000 nerve cells (less than 1% of total cells) from the Drosophila brain, and researchers spent a decade to collect data from 60,000 neurons at a rate of 1 gigabyte per cell. According to the same study, the human brain would take an estimated 17 million years [3] to collect the data from the 86 billion neurons using the same protocol. Furthermore, neural interface technology is trending towards wireless options as it has a significant advantage that the user can wear it for the long-term. Measuring neural activity throughout the day across a wide range of activities can provide a greater possibility for medical specialists to develop an effective patient-specific preventive solution. However, the more data these wireless neurotechnological devices gather, the more challenges we face, including high bandwidth, complex hardware, and more battery power required to handle the large amounts of data transferred from sensor end to decoder end of the wireless system.

As neuroscientists push the limits to understand the immense complexity of the brain with big data, researchers are developing efficient computational methods to handle and interpret the resulting data. Various data compression methods have been used for effective storage and transmission of multichannel electroencephalography (EEG) signals including, discrete cosine transform (DCT) based, discrete wavelet transform matrix (DWT) based, and run-length encoding. These methods need two independent steps, which are signal acquisition followed by compression. In contrast, compressive sensing (CS) allows data acquisition and compression simultaneously, which provide on-chip data compression and leads to efficient hardware implementation for signal acquisition. Therefore, CS has become an increasingly utilized paradigm for efficient data acquisition. The theory of CS was first introduced in [4] by Donoho et al. as a new paradigm for efficient data acquisition. The CS-based signal acquisition of an analog signal can be implemented with a continuous sensing operator that randomly sub-samples the input data and provides compressed measurements that consist of very few linear projections of the original signal. The theory of CS works well with the signals that can be represented by a significant *k* coefficients (i.e., *k*-sparse signals) over a *N*-dimensional basis. In other words, in contrast to the Nyquist–Shannon sampling theorem, CS relies on the sparsity data, and can be used for sampling the sparse signal below the Nyquist–Shannon limit, allowing its reconstruction from much fewer samples. In addition, CS offers synergistic time, and bandwidth reduction for wireless signal transmission with sparse sampling, and hence decreases the hardware and power requirements of the sensing devices.

The theory of CS was initially proposed for low-rate image acquisition. It was then developed for many other applications such as ultrasound imaging [5], face recognition [6,7], single- pixel camera [8], wireless sensors networks [9,10], cognitive radio networks [11,12], sound localization [13], audio processing [14,15], radar imaging [16,17], image processing [18,19], and video processing [20,21]. Similarly, CS has contributed to various neural engineering research including, neuronal network connectivity [22], magnetic resonance image (MRI) acquisition [23], MRI reconstruction [24], electroencephalogram (EEG) monitoring [25], compressive imaging [26,27], and other applications.

Furthermore, the CS framework has been also extensively used in Brain-Computer Interfaces (BCIs) in dealing with many challenges and to make current EEG-based approaches faster, and more energy-efficient. This paper provides a comprehensive review of the CS-related EEG studies by summarizing the related research papers published in the last two decades in terms of the CS reconstruction algorithms, the sparse basis, and the measurement matrix used in the BCI field. This work also highlights the current state of knowledge of the CS used in EEG studies and explains the possible challenges of the current BCIs are facing that lead to the development of effective future CS-based BCIs. Since a systematic review of CS for BCIs is missing in the literature, and this review will be useful for researchers and practitioners.

The rest of the paper is organized as follows. The motivation to use CS for BCI applications and an overview of currently available CS-based EEG studies are in Section 2. A detailed theoretical description of CS theory is discussed in Section 3, in which we also discuss the state-of-the-art about reconstruction algorithms, the sparse basis, and the measurement matrix being used in current BCIs. The idea of reconstruction free learning and related studies are discussed in Section 4. In Section 5, we provide the challenges and opportunities that future CS-based wireless neurotechnological devices may encounter. Finally, we conclude the review in Section 6.

## 2. Need and Applications of CS for EEG

**Figure 1:**
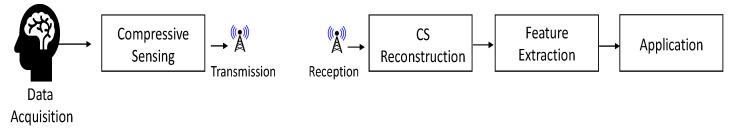
Block diagram of CS-based EEG studies

With recently growing progress in neural engineering research, EEG based methods have been revealed to be a promising approach for diagnosis, rehabilitation, and restoration of motor functions. In wireless EEG based devices, batteries have been used as the primary source of energy. Sophisticated tasks in practical wireless BCI applications—including signal acquisition, signal processing, and wireless EEG transmission for long term—result in heavy power consumption. Furthermore, the data recorded from multi-channel neural recording implants and neurological devices result in large storage space. The CS-based EEG compression and sensing can provide saving in signal acquisition, and transmission to facilitate signal processing in the resource constraints circumstances. Roughly, the general CS-based EEG framework can be broadly divided into four main steps (shown in Figure 1), which are signal acquisition and compression at the sensor node, and signal reconstruction, feature extraction, and decoding at the receiver node.

In this regard, the researchers around the world are working on improving these four steps and proposed various interesting applications. For instance, R. Meenu et al. [28] used the idea of CS for detecting the presence or absence of epileptic seizures in the EEG signal. The work proposed by K. Zeng et al. [29] used the compressibility calculated using the CS theory in EEG for automatic detection of seizure states. The authors used continuous wavelet transform (CWT) to extract features from time-frequency representation, related to ictal, pre-ictal, and ictal EEG. T. Moy et al. [30] proposed CS-based EEG signal acquisition and biomarker-extraction system with flexible, thin-film electronics. They successfully demonstrated the reconstruction of compressed EEG signals at up to 8× compression and spectral feature extraction for seizure detection from compressively sampled EEG signals. Another study by A. Abdulghani et al. [31] discussed the trade-offs related to the practical performance of CS for long term EEG signals. The work proposed by K. Abualsaud et al. [32] also utilized the idea of CS in wireless EEG-based epileptic seizure detection and investigated the trade-off between the complexity of CS-based framework in terms of power and classification accuracy. A few other studies [33,34,35] also used the theory of CS for seizure detection and showed a significant reduction in computational power and storage.

Furthermore, to address the issue of the massive amount of data originating from EEG acquisition, the study proposed by N. Mammone et al. [36] also utilized the theory of CS to compare the characteristics of the brain network organization in Alzheimer’s disease (AD), mild cognitive impaired (MCI), and healthy elderly subjects by analyzing their EEG recordings. F. Morabito et al. [37] also proposed an interesting study on finding the compressibility property of EEG signals collected from AD patients, MCI patients, and healthy elderly. The finding of this study shows that it is possible to use the CS framework to compress and reconstruct the EEG signal with the signal quality required by clinical constraints, which not only reduces the throughput but also discriminate brain states among AD, MCI, and normal healthy elderly. A hardware implementation of a low power multichannel EEG signal acquisition system based on distributed CS is proposed by B. Kaliannan et al. [38], which enables lower power and lesser complexity in the sensing end. In order to reduce the power consumption and area overhead, M. Hosseini et al. [39] also proposed a new microelectronic system for compressive recording of multichannel high density intracranial neural signals. The performance of efficiently reconstructed multi-channel neural signals in this study is evaluated through the system and circuit-level simulations. As another application of CS in BCIs, R. Shriwastav et al. [40] proposed a novel BCI based on CS and a deep learning framework, where the authors reconstruct the motor imagery EEG signal using a convolutional neural network (CNN). The study proposed by H. Lee et al. [41] presented an automatic sleep-stage classification system by utilizing CS for EEG data compression. This work used a radial basis function (RBF) neural network for sleep-stage classification utilizing the temporal statistical and spectral features extracted from the reconstructed EEG signal. The results of this study showed that the CS-based approach achieved high classification accuracy and reduced the hardware complexity of the system with respect to the previously reported sleep-stage approaches.

## 3. CS for EEG Signal

As discussed before, CS randomly sub-samples the input data and directly generates compressed measurements while still preserve the information of interest. To understand CS mathematically, consider x be the signal of interest of dimension N×1. A measurement vector y can be computed using an M×N matrix Φ. Mathematically, the signal sampling model using the CS framework can be given by (1)

(1) y=Φx,

where, $x\;\epsilon \;R^{N}$ is the input signal, $\Phi \;\epsilon \;R^{M\times N}$ is the CS measurement matrix and $y\;\epsilon \;R^{M}$ is the compressed measurements. Furthermore, *N* represents the number of samples in the input signal, and *M* represents the number of measurements. In a typical CS setting M<<N, so that there are fewer measurements in y than the original signal x.

Here, it is important to emphasize that certain conditions should be fulfilled for an efficient CS scenario, which includes input signal sparsity and incoherence between Φ and the sparsity basis. Important methodological considerations for CS such as sparse representation, sensing matrix design, and reconstruction algorithms will be covered in detail in the following sub-sections.

### 3.1. Sparse Representation

In Equation (1), the recovery must incorporate some prior knowledge on original signal x. The structure that is widely assumed in CS is sparsity, and, therefore, the theory of CS relies on the assumption that the signals being processed are sparse in nature. A signal x is called *k*-sparse if it has only a few nonzero entries (i.e., *k* nonzero coefficients). However, for several practical applications, signals such as EEG are not explicitly sparse but near sparse. If the signals of interest are not sparse enough, it can be sparsified with respect to such overcomplete dictionaries Ψ into its transformed domain; this is known as a dictionary or sparse basis. These dictionaries obtain the sparse form of the signal by transforming it into other domains, such as wavelet, Gabor, splines, and Fourier domain. The dictionary is a fixed matrix, which maps the input signal as a sparse linear combination of its elementary signals called "atoms". These dictionaries are highly redundant, which allows us to obtain a sparser representation with a variety of atoms. A study by D. Wen et al. [42] discussed more on sparsity of an EEG signal and presented a review of sparse representation-based EEG classification methods for epilepsy detection, BCIs and cognitive impairment.

Assume that a one-dimensional discrete-time signal s of length *N* exhibits sparsity in certain orthonormal basis Ψ defined by the basis vectors $\Psi =[\Psi_{1}|\Psi_{2}|\Psi_{3}|\cdots \Psi_{N}]$. Therefore, the signal s can be represented using its sparse transform domain vector x as follows:

(2) $$x=\Psi s=\sum_{i=1}^{N}s_{i}\Psi_{i},$$

where s is the N×1 column vector of the signal of interest. Here, it is clear that both x and s are different representations of the same signal with time and Ψ domains, respectively. The value of x in (1) can be replaced by Ψs using (2), and, therefore, it is obtained

(3) y=Φ(Ψs),

(4) y=As,

where A in (4) is a M×N dimension matrix, which transforms and compresses a *k*-sparse signal x into a M×1 measurement y.

In the last few years, researchers have devoted lot of effort for developing a suitable dictionary that helps to find the sparser representation of the EEG signal. In the literature, the Gabor dictionary, which can be defined by Gaussian envelope sinusoidal pulses [31] is widely used as a sparse basis. For instance, the work proposed by M. Mohsina et al. [43] and S. Aviyente et al. [44] uses Gabor transform as the sparsifying basis of EEG with the focus of reducing energy consumption for processing and transmission. However, they did not consider the inter-channel correlation of the EEG signal in their study. P. Dao et al. [45] evaluated the effect of different time and frequency step sizes in building Gabor atoms [43,44] on EEG signal compression using CS. Furthermore, P. Dao et al. [46] also discussed on a quantitative comparison of CS using Gabor and K- singular value decomposition (SVD) dictionaries. Similarly, R. Kus et al. [47] also proposed a novel construction of an optimal Gabor dictionary for multichannel and multi trial EEG, which allows a priori assessment of maximum a one-step error of the matching pursuit (MP) algorithm. The work proposed by H. Zhang et al. [48] also used the over-complete Gabor dictionary for the EEG signal. Few researchers, such as L. Chen et al. [49] and R. Quian et al. [50] also used the Gabor coefficients of the EEG signals for seizure detection. Furthermore, F. Morabito et al. [37] used the classical Gabor wavelet dictionary for the dictionary functions for Alzheimer’s disease (AD) analysis. They claimed that a joint use of different channels with the same approach could further improve the compressibility of EEG signals from AD patients, as AD is also known to be responsible for perturbed synchrony among channels. However, a technical issue still persisting for most of Gabor dictionary is the over complete redundant representation.

The discrete cosine transform as a dictionary basis is another commonly used approach. The study proposed by Z. Zhang et al. [51] used the idea of DCT and inverse DCT matrix as the dictionary matrix Ψ to find the sparse representation of the EEG signal. In this study, the authors compared the performance of the DCT dictionary Ψ in combination with a few representative CS reconstruction algorithms. Another research conducted by D. Birvinskas et al. [52] also used DCT domain representation by expressing an EEG signal as a DCT coefficient vector for signal compression. The Mexican hat function defined by the second derivative of Gaussian functions [45] as a dictionary basis is also used to find the sparse basis for the time-frequency analysis of EEG signals [53].

The research growth in the above-mentioned various sparse basis for the EEG signal offers researchers an opportunity to explore more on EEG signal sparsity. However, these methods have difficulty in reconstructing accurate EEG signals because of the apriori assumption of signal structures, and fixed basis function selection. Recently, data-driven dictionary learning has been explored to represent EEG signals into its sparse domain, which is more suitable for the sparseness of neural activity signals such as EEG. The work proposed by M. Fira et al. [54] proposed a data-driven dictionary design by building the dictionary using the EEG signal from the training dataset itself rather than using any fixed dictionary. Furthermore, apart from these mainstream dictionaries for EEG signal, few other studies also used B-Spline dictionary [55,56], linear and cubic-Spline dictionaries [57], Spline dictionary [58], Meyer wavelet function dictionary [59], or Daubechies wavelets function dictionary [60] to obtain the sparse representation of the input signal.

### 3.2. Sensing Matrix

**Figure 2:**
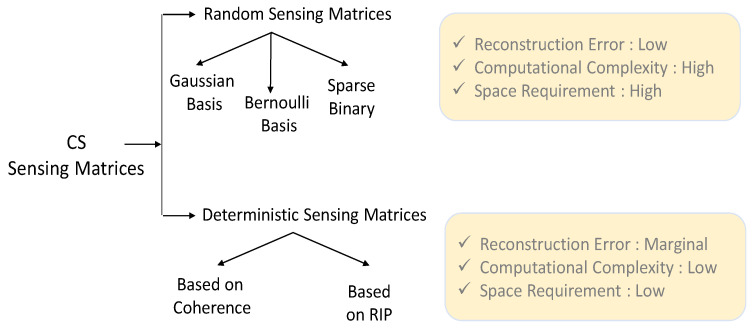
Classification of sensing matrices

An efficient design of suitable Φ is one of the important aspects of the CS framework because Φ plays an important role for the compression of x at the sensor end and its reconstruction from y at the receiver end with given Φ and Ψ. A variety of sensing matrices such as random sensing matrices, deterministic sensing matrices, and structural sensing matrices are proposed in the literature. Figure 2 shows a broad classification of available sensing matrices. However, most of the sensing matrices are designed with the assumption that the signal x is explicitly sparse, which is not the case for the EEG signal.

A primary criterion for CS success is the incoherence between the sampling basis Ψ and the sensing basis Φ, i.e., Φ must be maximally incoherent with the sparsifying dictionary Ψ, and, therefore, the researchers have focused on designing the sensing matrix Φ to be a random matrix so that it becomes nearly orthogonal. To this end, various random distribution functions, such as Gaussian or Bernoulli distributions are used to design a random sensing matrix Φ.

In the context of the EEG signal, the Gaussian random matrix is commonly used as a CS signal sensing matrix in the literature. Many studies [31,36,38] used the Gaussian random matrix Φ in their work. In the study proposed by J. Zhu et al. [60] a new algorithm towards CS reconstruction of multichannel EEG signals by exploiting cosparsity and the low-rank property also used Gaussian random matrix as a sensing matrix. Recently, S. Qiu et al. [61] proposed a CS framework for BCIs based on teleoperation control of robotic exoskeleton using steady-state visual evoked potentials (SSVEP). They used Gaussian random matrix in their integrated study of CS, various brain–machine reference commands, and adaptive fuzzy controllers together. The study proposed by H. Liu et al. [62] discussed various random matrices including Gaussian random matrix for resting-state EEG signal classification of schizophrenia. Although Gaussian random matrix is commonly used for measurement sensing, it is not suitable for wireless neural engineering applications because a Gaussian random generator would lead to a large number of matrix-vector multiplications (energy-intensive), and therefore, it cannot be efficiently implemented on a signal acquisition hardware [63]. In order to make the sensing process faster and less computationally complex, few studies [29,35] used a full Bernouilli matrix, as it is easier to generate its random entries with fewer multiplication operations. However, it is still a challenge to use Bernouilli matrix due to its space power consumption in hardware. An alternative to this problem could be the sparse binary sensing matrices, which only contain a few nonzero (one’s) entries in each column of the matrix. An interesting study proposed by J. Sheng et al. [64] compared the performance of the random Bernouilli matrix and sparse binary sensing matrix and concluded that sparse binary sensing matrix are often a better choice over random Bernouilli matrix for implementing CS for the wireless body sensor network (WBSN). A comparative performance evaluation study proposed by R. Tello et al. [65] on an independent SSVEP-BCI based on CS also used a random sensing matrix in their experiment. This study focuses on the detection of visual attention in SSVEP-BCIs for people with severe motor disabilities. In order to reduce the matrix multiplication operation further, A. Gilbert et al. [66] also used a sparse binary sensing matrix. Although the reconstruction performance of sparse binary sensing matrices is not good enough in comparison to full random matrices, it has been used in many other studies such as [36,39,51,63,67,68]. N. Mammone et al. [36] adopted the sparse binary matrix as a measurement matrix, inverse DCT matrix as a dictionary matrix to compress and, subsequently, reconstruct the high-definition (HD)-EEG signals obtained from AD, MCI and healthy subjects. The work proposed by M. Fira et al. [54] also used a sparse binary sensing matrix in their experiments. They also compared the reconstruction error for four different sensing matrices, which are: Gaussian random matrix, Bernouilli random matrix, a random sparse binary sensing matrix, and a fixed sparse binary sensing matrix. In conclusion, they recommended using a sparse binary sensing matrix for future wireless BCI applications. Furthermore, M. Hanafy et al. [69] proposed a study for ZigBee-based EEG telemonitoring and used a binary sensing matrix in their design. Similarly, A. Majumdar et al. [70] also suggested the use of binary sensing matrix to reduce the sensing power consumption in their energy efficient CS-based EEG sensing design.

Numerous research has demonstrated the effectiveness of the aforementioned random sensing matrices. However, the random sensing matrices have high reconstruction complexity, and computationally demanding, which need significant space requirement for storage. In order to go beyond these limitations, there is a growing interest for developing deterministic sensing matrices. T. Nguyen et al. [71] investigated and presented a good review of various deterministic sensing matrices. Furthermore, few research, as H. Monajemi et al. [72], also claimed that randomness of the matrix Φ, such as Gaussian random matrix, is not required for the phase transition phenomenon with the assumption that the positions of the non-zeros are chosen purely at random. The system complexity of the random dense matrices-based approach is typically $O(N^{2})$, whereas implicit operations often can be carried out with the system complexity of O(Nlog(N)) using such deterministic sensing matrices [72]. These deterministic sensing matrices do not require explicit data saving space and memory accesses and, therefore, these can be used for designing future highly miniaturized wearable BCIs.

As discussed, the deterministic sensing matrix can make the hardware realization convenient and easy for the considered applications, hence, deterministic sensing has been widely investigated in the literature and already been used in many other applications [73,74]. The deterministic sensing matrix has been exploited for BCI application. For example, W. Zhao et al. [75] used deterministic quasi-cyclic array code (QCAC) matrix-based compressed sensing encoder architecture for wireless neural recording applications. Further, W. Zhao et al. [76] also proposed the construction of the QCAC matrix and sparse random binary matrix (SRBM) and performed simulation experiments to reconstruct EEG signal from compressed measurement. The authors also compared the reconstruction performance of QCAC, SRBM, and random binary matrix and showed that these matrices are energy-efficient and had considerable reduction in computational resources.

### 3.3. Reconstruction Algorithms

**Figure 3:**
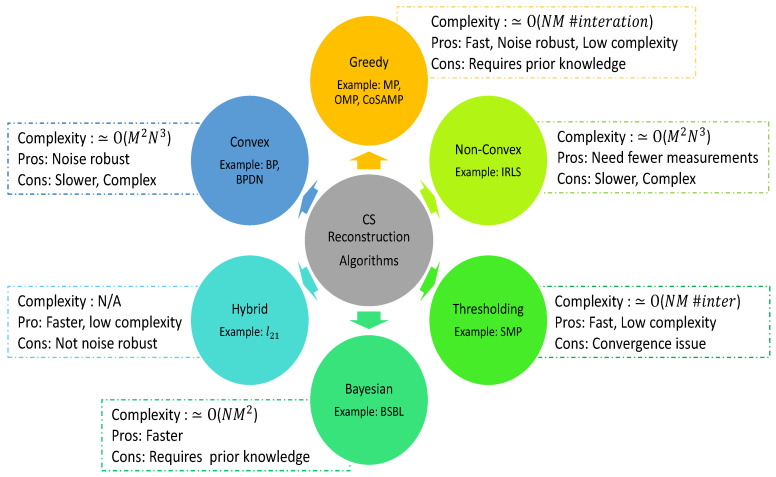
Classification of reconstruction algorithms.

It is interesting to note that the signal acquisition, compression and transmission of CS measurements are relatively easy and it is the exact reconstruction of the signal that is complex. On the contrary, it is reverse for the classical sampling based on the Nyquist theorem. A class of CS reconstruction algorithms has been developed and applied to recover the original signal from the compressed measurement of y. The reconstruction algorithms available in the literature can be broadly divided into six categories, as shown in Figure 3. At the receiver end, the CS reconstruction algorithms basically seek to find an exact solution of an underdetermined system of (1) from infinitely many solutions. These algorithms are usually iterative and pursue a key goal to reduce the error between the original and the recovered signal.

In the context of EEG signal for BCI application, mostly nonlinear algorithms are used, which require prior knowledge of sparsifying Ψ and A. Basically, the recovery of s in (3) from *M* compressed measurements, seeks for solution $\hat{s}$ by finding minimum few non-zero entries for an under-determined system N>>M. Mathematically, the recovery of s using $l_{0}$-minimization is formulated as:

(5) $$\hat{s}=\underset{\hat{s}}{\arg\;\min}{∥\hat{s}∥}_{l_{0}}\mathrm{subject}\;\mathrm{to}:y=A\hat{s},$$

where ${∥\hat{s}∥}_{l_{0}}$ is the number of non-zero entries in s. A limitation of (5) is that it is NP-hard and ill-conditioned because of the non-convex nature of $l_{0}$-minimization. However, (5) can be reformulated into a convex problem and a unique solution can be found by using $l_{1}$-minimization given by (6)

(6) $$\hat{s}=\underset{\hat{s}}{\arg\;\min}{∥\hat{s}∥}_{l_{1}}\mathrm{subject}\;\mathrm{to}:y=A\hat{s},$$

where ${∥\hat{s}∥}_{l_{1}}=\sum \left|s_{i}\right|$.

The advantages of $l_{1}$-minimization is its flexibility to incorporate prior information into signal reconstruction model and its uniform recoverability. A convex optimization problem that finds a solution having a minimum $l_{1}$-norm is often called basis pursuit (BP), which is a commonly used approach for signal reconstruction. For instance, F. Morabito et al. [37] used $l_{1}$-norm and proposed an interesting study on finding the compressibility property of EEG signal collected from AD patients, MCI subjects and healthy elderly. Similarly, M. Fira et al. [54] used $l_{1}$-norm for EEG signal reconstruction for the analysis of the specific dictionaries of EEG signals. Furthermore, M. Fira et al. [77] also used $l_{1}$-norm in their study and proposed CS based framework for the P300 detection spelling paradigm.

The $l_{l}$-minimization techniques are powerful methods for CS signal recovery when dealing with noise-free measurements. However, in the case of noisy measurements, it may show a poor recovery performance and this issue can be handled using $l_{2}$ norms. Similar to $l_{1}$ norm, $l_{2}$ norm optimization can be formulated as

(7) $$\hat{s}=\underset{\hat{s}}{\arg\;\min}{∥\hat{s}∥}_{l_{2}}\mathrm{subject}\;\mathrm{to}:y=A\hat{s},$$

where ${∥\hat{s}∥}_{l_{2}}=\sum {\left|{s_{i}}^{2}\right|}^{1/2}$.

In BCI research, few studies adopted $l_{2}$ norm approach to reconstruct the EEG signal. For example, the study proposed by T. Moy et al. [30] for acquisition and biomarker-extraction system used $l_{2}$ norm for EEG signal reconstruction. Another work proposed by K. Abualsaud et al. [32] also used $l_{2}$ norm for EEG-based epileptic seizure detection. In most BCI applications, $l_{1}$ and $l_{2}$ based approach showed promising results. However, these norms suffer from significant limitations, as they do not take temporal dynamics of the EEG signal into account, they fail to recover the time courses of EEG data. In order to address this limitation, few researchers such as A. Gramfort et al. [78] used combinatorial optimization or mixed norm of both $l_{1}$ and $l_{2}$, also called $l_{21}$ norm optimization for Magneto-EEG signal. Similar to [78], $l_{0}$ norm can be used with other norms to efficiently solve the optimization. For example, Y. Liu et al. [79] used a combinatorial optimization with the $l_{0}$ norm and Schatten${}_{0}$ ($s_{0}$) norms to encourage cosparsity and low-rank structure in the reconstructed EEG signals. Further, the alternating direction method of multipliers (ADMM) is used to solve the simultaneous cosparsity and low-rank (SCLR) optimization. The study proposed by J. Zhu et al. [60] also proposed an algorithm towards CS reconstruction of multichannel EEG signals via $l_{q}$ norm and $s_{p}$ norm regularization. They make use of $l_{q}$ norm and $s_{p}$ norm to enforce cosparsity and low-rank property in the reconstructed EEG signal. Further, they applied ADMM to the resulting nonconvex optimization and compared the performance of $l_{q}$ norm and $s_{p}$ with other competitive reconstruction algorithms and claimed superior performance.

Furthermore, M. Tayyib et al. [80] proposed an accelerated sparsity-based reconstruction of compressively sensed multichannel EEG signals using ADMM. First, they obtained the $s_{p}$ norm along with the decorrelation transformation of EEG data, and then double temporal sparsity-based reconstruction algorithm has been applied for the signal reconstruction. Few other recent studies [81] have also shown that combining cosparsity and low-rank property usually results in efficient CS reconstruction of multichannel EEG signals. However, these studies rarely incorporated the effect of noise in their studies. It is worth commenting that the noisy measurements may degrade the performance of CS reconstruction algorithms. The study discussed by X. Zouab et al. [82] proposed a robust CS reconstruction algorithm called regularized cosparsity and low-rank property (RCS-CLR) to accurately recover multichannel EEG signals from noisy measurements in the presence of impulsive noise. Furthermore, most of the cosparsity based methods ignore the adjacent relationship between the real physical electrodes and use convex regularizations to exploit cosparsity and channel correlation. This may also degrade the performance of the reconstruction method. In order to enforce inherent correlation across different channels and cosparsity of multichannel EEG signals, X. Zou et al. [81] proposed a graph Fourier transform and nonconvex optimization (GFTN)-based method, which can exploit the accurate adjacent relationship between the real physical channels. Similar to [80], this work also used ADMM for signal reconstruction.

The Bayesian-based method is another commonly used approach for signal reconstruction from compressed measurements. The block sparse Bayesian learning (BSBL) was initially introduced by Z. Zhang et al. [51] as an alternative CS reconstruction algorithm and achieved promising results for telemonitoring of nonsparse signals such as EEG. The work proposed by K. Zeng et al. [29] and N. Mammone et al. [36] also adopted the BSBL algorithm as EEG reconstruction method. The work proposed by S. Fauvel et al. [63] exploits both temporal and spatial correlations to efficiently compress EEG signals in WBSNs and used BSBL based approach for EEG reconstruction. In this study, the authors compared the energy consumption (for cycle count, run time, computation and computation plus transmission) and reconstruction accuracy of the various framework on a wide range of EEG applications. The BSBL algorithm showed satisfactory reconstruction performance for various applications. Particularly, for reconstruction of multichannel EEG signals, it is time-consuming. To address this issue, Z. Zhang et al. [83] proposed a spatiotemporal sparse Bayesian learning algorithm to reconstruct multichannel signals simultaneously for BCIs and EEG-based driver’s drowsiness detection by exploiting temporal correlation within each channel and inter-channel correlation among different channel signals. Furthermore, H. Mahrous et al. [68] proposed a novel method for compressing multi-channel EEG signals by exploiting both linear and non-linear dependencies in the EEG data. This study enables the conventional BSBL-BO to produce a better reconstruction of the EEG signal because of significantly better “block” sparsity structure than the state-of-art BSBL-BO. B. Liua et al. [84] proposed a fast BSBL (BSBL-FM) reconstruction algorithm CS framework. They also implemented BSBL in FPGA and compared the performance of CS-based and wavelet-based compression algorithms in terms of power and energy consumption.

As discussed before, BP uses $l_{1}$ norm minimization to solve the optimization problem. Greedy algorithms pursue a similar goal as convex algorithms to minimize the error between the original signal and the recovered signal. In the BCI literature, many variations of BP are proposed. For example, H. Lee et al. [41] presented an automatic sleep-stage classification system and used the orthogonal matching pursuit (OMP) algorithm for EEG signal reconstruction. Similarly, B. Kaliannan et al. [38] proposed a hardware realization of the multichannel EEG signal based on simultaneous orthogonal matching pursuit (SOMP) and theory of joint sparse recovery for EEG reconstruction. The study discussed in [85] also utilized basic SOMP along with other algorithms for distributed CS of jointly sparse signals. Furthermore, in order to deal with non-sparsity on the EEG signal, H. Djelouat et al. [86] used subspace pursuit algorithm with the concept of a concatenated basis where a sparsifying basis consists of randomly selected atoms from both DCT and DWT. The work proposed by R. Kus et al. [47] provides more mathematical details on OMP properties and its advantages, and proposed multivariate matching pursuit for EEG signal reconstruction. Furthermore, basis pursuit denoising (BPDN) is an another computationally efficient approach used in the BCI literature to reconstruct the EEG signal. For instance, the study proposed in M. Hosseini et al. [39] have used BPDN with a joint sparse decoding algorithm for reconstruction of multichannel intracranial neural data and X. Li et al. [67] used BPDN to understand the effect of epoch length on CS reconstruction. In the literature, various other reconstruction algorithms are also proposed for specific applications, such as A. Khoshnevis et al. [87], which used a novel reconstruction method for event-related potential signals using Kronecker approach to improve the quality of reconstruction and accelerate the compression phase. In the study proposed by R. Shriwastav et al. [40], the convolutional neural network (CNN) is used to reconstruct the motor imagery EEG signal.

In addition to the aforementioned studies, few research has been focused on comparing the reconstruction performance of various approaches proposed. For example, X. Zouab et al. [82] compared the performance of RCS-CLR with state-of-the-art methods such as ADMM-based SCLR (SCLR-A) [78], BSBL [51], and SOMP [38] and showed a superior reconstruction performance in the presence of noise. In the study by Z. Zhang et al. [88] reported a comparison result of twelve typical sparse signal recovery algorithms such as compressive sampling matching pursuit (CoSaMP), hard thresholding pursuit (HTP), subspace pursuit, among others for EEG signals. Similarly, A. Abdulghani et al. [31] compared the performance of 18 different combinations of BP, MP, and OMP with six dictionary matrices (Gabor, Mexican hat, spline- linear and cubic, and B-spline- linear and cubic) for long term scalp EEG signals. The experiments performed by M. Rani et al. [89] compared the performance of the CS reconstruction algorithms such as BP, BPDN, OMP, and CoSaMP for determining a better reconstruction of EEG signal, and concluded that if reconstruction speed is a prime concern, then OMP is a better choice, whereas, for higher reconstruction quality, CoSaMP is a preferred option. Furthermore, R. Tello et al. also compared the classification performance of EEG signal reconstructed using three algorithms namely BSBL-BO, $l_{p}^{d}$- regularized least-squares ($l_{p}^{d}$-RLS) and $l_{p}^{2d}$-RLS. The results in this study reflect that $l_{p}$-RLS based reconstruction approach shows better performance compared to the BSBL-BO. The effectiveness of the improved $l_{p}$-RLS reconstruction approach is also demonstrated by D. Gurve et al. [90] for other applications such as fetal electrocardiogram (ECG) extraction from abdominal ECG signal.

A summary of the detailed empirical comparison of selected state-of-the-art CS-based BCI methods in terms of reconstruction algorithms, sensing matrix, and sparse basis used is shown in Table 1 and Table 2.

## 4. Reconstruction Free CS

As discussed in earlier sections, a number of CS algorithms are being actively pursued in the field of neural engineering, however, to satisfy the constraints of many practical BCI applications it is essential to minimize the computational complexity of the CS reconstruction algorithm. In contrast, the high computational complexity of available CS reconstruction algorithms is an obstacle for many real-time applications. For instance, for basis pursuit and CoSAMP, the computational complexity is $O(N^{3})$ and O(M, N), respectively. Few studies [91,92,93] discussed on the computational complexity of various other CS reconstruction algorithms in detail.

**Figure 4:**
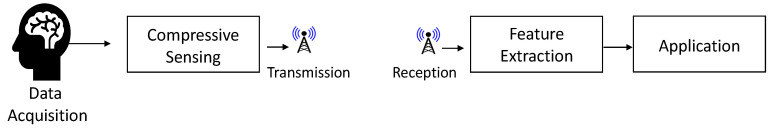
Block diagram of reconstruction free CS-based EEG studies.

Interestingly, for some classification applications, we may not require an accurate reconstruction signal of interest to extract the features for classification. In these cases, it may be possible to perform feature extraction and classification directly on the compressed measurements, without reconstructing the signal of interest. More specifically, different from the previous traditional CS, the signal of interest can be compressed using CS at the sensing node and the features can be extracted directly from compressed measurements at the receiver end. This reconstruction free CS approach is called “compressive feature learning”. Figure 4 shows the reconstruction-free compressive learning CS framework. In this section, we take an in-depth look at the currently available reconstruction free CS approaches in the developing field of BCIs from the perspective of the machine learning classification and system complexity.

Motivated by the fact that meaningful features can be extracted from the compressed measurement directly, many researchers have focused on reconstruction-free compressive learning for various applications such as surveillance and autonomous navigation [94], compressive cameras [95], and text data classification [96]. Furthermore, Lohit et al. [97,98] and Braun et al. [99] also hypothesize that the reconstruction step can be entirely bypassed, and shown the optimal machine learning performance for computer vision applications, using reconstruction-free compressive learning. In the context of neural engineering, very limited but successful attempts have been made towards the feature extraction from the compressed domain. For instance, M. Shoaib et al. [34] proposed a seizure-detection system that directly uses compressively sensed EEG measurements for embedded signal analysis. In particular, they proposed an algorithm and a hardware architecture that enables two power-management knobs to quantify the amount of data compression and to determine the approximation error for the compressed-domain analysis. Similarly, M. Shoaran et al. [35] proposed a multichannel compressed-domain feature extraction as a low-power technique for data compression and seizure detection from multichannel cortical implants. This work also shows the success of using compressed feature learning, which reduces the computational energy for analysis due to a reduction in reconstruction costs. In order to leverage the benefits of compressed-domain feature extraction, R. Aghazadeh et al. [14] also proposed a new algorithm for epileptic seizure detection using a compressively sensed multichannel EEG signal. S. Qiu et al. [61] utilized the concept of feature extraction from the compressed measurements and proposed a teleoperation control system for robotic exoskeleton performing manipulation tasks. This work conveys the control signals to the robotic exoskeleton using features extracted directly from compressed SSVEP measurements. Furthermore, M. Fira et al. [100] also proposed EEG signals classification for a spelling paradigm in the compressed domain.

In summary, the main advantage of the reconstruction free CS approach is that with the feature extraction from compressed measurements directly, the reconstruction step at the receiver node can be entirely bypassed and, therefore, greatly reduces the computation complexity of the system. Additionally, the reconstruction free CS approach not only reduces the system complexity but also reduces the processing time at the receiver end. On the other hand, the reconstruction free CS is an analysis framework and signal synthesis is not a desired outcome.

## 5. Discussion and Future Directions

Neuroscience is a fascinating field and has a profound impact on people’s health. The possible technologies that to be integrated with future neural interface technology certainly have the potential to improve our health even more. We are probably standing on the threshold of a complete transformation of the neuroscience industry by the use of big brain data from clinical and consumer-directed neurotechnological devices, which may provide reduced latency and permit a real bidirectional interaction with our brain. Furthermore, the applications of neural implants in the current neuroscience industry are still in their infancy, but we are likely to see in the future functionalities of a neural interface, starting from brain-to-brain connectivity, cell level the neural activation of the different channels, personalized treatments in patients affected by chronic neurological diseases to monitor the production of neurotransmitters, direct stimulation of neurons and bidirectional brain-to-machine interoperability in the forthcoming years.

The CS-based wireless neurotechnological devices can address the challenges encountered in a low power application where sensing power is very critical such as battery-operated EEG sensing implants. However, in CS for neurological signals, there is no “bread and butter” solution, which means no one algorithm works well for every problem and cannot be assured for all the applications. This is widely applicable at the receiver end of CS where we reconstruct the original signal using various algorithms. As a result, one should use a different algorithm for different applications, which would lead to optimum results based on the nature of the signal used in the BCI framework. However, in a more practical way, it can be concluded that BSBL and BPDN are a more suitable choice for most applications, where accurate reconstruction is needed, and computation time is not of primary concern. However, for most of the real-time applications, thresholding-based reconstruction could help where the computation time is not of primary concern. Furthermore, BPDN outperforms OMP and BSBL in case of noisy measurements for accurate reconstruction. A similar conclusion can be drawn for the BCIs where computational complexity is a design concern. In such case, reconstruction algorithms such as OMP is preferable. However, the performance of any reconstruction algorithms depends on many other parameters. For instance, X. Li et al. [67] investigated the effect of EEG signals epoch length on the CS reconstruction algorithm and concluded that a longer epoch length leads to better signal compression at the expense of larger signal reconstruction time. The findings in this paper shows that at the sampling frequency of 256 Hz, a 4-s epoch length is suitable to perform the signal reconstruction. However, the signal reconstruction performance also depends on the performing hardware platform not just on the epoch length of an EEG signal. Furthermore, apart from the accurate reconstruction of the EEG signal, the accurate identification of single-unit neural activities (spikes) has a key role in developing high-accuracy BCIs. To this end, various works have been done such as dynamic evolving SSNs [101] and spiking neural networks (SNNs) [102] and demonstrated that an SSN does a strong compression of the EEG signal when it is coded as spikes. In context of CS for BCIs, [40] also showed that spike signals are highly compressed using the CS technique with up to a 90% compression rate.

Recent progress has suggested how CS can help to optimize sensing resources, transmission, and storage capacities, as well as to facilitate signal processing in an energy constraint BCI environment. Despite the attention that CS has received in recent years in various fields of neural engineering, few limitations still reside. We conclude this review by raising the following five major challenges and opportunities to be addressed in the future of big data processing, which will be aided by further developments in the CS field.

- Despite of recent advances in hardware technologies and the feasibility of performing on-chip signal processing, most of BCI research reviewed in this article demonstrated the applicability of CS framework with digital implementation, which relies on many assumptions. In the future, it is expected to see more analog implementations of CS to minimize energy consumption under real-life conditions with built-in hardware.
- Although random matrices are widely used for CS-based BCIs and ensure high reconstruction accuracy, storing random matrices requires a lot of memory. This disadvantage of the random sensing matrix can be addressed through their deterministic construction. The advantages of the deterministic matrix are simplicity in sampling and reconstruction stages, and reduced computational complexity. In the future, it would also be interesting to develop strategies for CS measurements under a deterministic sensing matrix for wireless BCIs.
- In the study proposed by F. Chen et al. [103], a hardware-efficient circuit model was developed for the analysis of analog and digital implementations of the CS-based EEG compression model. The analysis in this study reveals that a digital implementation of CS is a significantly more energy-efficient and suitable architecture with respect to their proposed analog implementation. In future research, an efficient analog implementation of CS needs to be explored more for next-generation CS-BCI applications.
- The advancements in the acquisition and processing of large data sets in the field of neural engineering is permitting a greater understanding and clinical observations of patients with brain disease. However, most of the current research has not yet achieved a personalized data-driven approach for treatment. In other words, the current research lacks in terms of developing a quantitative integrative tool to translate these understanding and clinical observations to the individual level to build the basis for personalized treatment. This is an important area which should be considered for future studies.
- The CS and BCI systems have created a hope for the implementation of many practical applications, but as data volumes go up, and BCI research are moving towards the creation of computationally demanding algorithms for more complex applications, CS would not help for real-time applications. In such case, reconstruction free learning could help for many machine learning-based applications. In future, such reconstruction free learning will be more highlighted for many other BCI applications.

The CS is an energy-efficient approach for future BCIs application, which has been developed over the past few years. However, there are still numerous BCIs application areas in which the CS theory continues to be developed including rehabilitation and restoration [104,105], communication and control [106,107], prevention [108,109], and user state monitoring [110,111]. Although it is beyond the scope of this particular review article, security [112,113] and entertainment [114,115] are other very interesting topics in the BCI field, which can be benefited with CS and should be considered for future studies.

## 6. Conclusions

The CS framework can help in dealing with many challenges that current BCIs may encounter, which requires the use of fast, long-term, and energy-saving computational approaches. In this article, we have reviewed the available BCIs, which have benefited from the theory of CS in terms of sparse signal representation, sensing matrix, and available reconstruction methods. The discussion around multiple CS reconstruction algorithms reviewed in this article gives us a more complete landscape of this problem as different algorithms come with different trade-offs, such as time and computational complexity. We have also highlighted the advantages and disadvantages of the available algorithms to explore these trade-offs. Though the main focus of this article is to study CS and related BCIs available so far, the paper nevertheless also discusses the idea of reconstruction free CS used for various applications to reduce the complexity of the system. 

## Figures and Tables

**Table 1:** A summary of CS applied EEG studies (application focused).

Studies	Applications	Reconstruction Algorithms	Sensing Matrices	Sparse Basis
A. Abdulghani et al. [31], 2012	Seizure detection	BP, MP, OMP	Gaussian random matrix	Gabor dictionary
K. Abualsaud et al. [32], 2013	Seizure detection	$l_{2}$ norm minimization	Random matrix	DCT dictionary
F. Morabito et al. [37], 2013	Alzheimer’s disease analysis	$l_{1}$ norm minimization	Gaussian random matrix	Gabor dictionary
M. Shoaib et al. [34], 2014	Seizure detection	Reconstruction free	Random matrix	-
B. Liua et al. [84], 2014	Seizure detection	BSBL-FM	Sparse binary matrix	Wavelet dictionary
Z. Zhang et al. [83], 2014	SSVEP & Drowsiness detection	STSBL-EM	Sparse binary matrix	DCT dictionary
K. Zeng et al. [29], 2016	Seizure detection	BSBL	Bernoulli random matrix	Gabor dictionary
M. Fira et al. [54], 2016	P300 spelling	$l_{1}$ norm minimization	Random matrix	Wavelet dictionary
M. Fira et al. [77], 2016	P300 spelling	$l_{1}$ norm minimization	Random matrix	Data driven dictionary
M. Shoaran et al. [35], 2016	Seizure detection	Reconstruction free	Bernoulli binary matrix	-
T. Moy et al. [30], 2017	Seizure detection	$l_{1}$ norm minimization	Random matrix	Gabor dictionary
R. Aghazadeh et al. [33], 2018	Seizure detection	Reconstruction free	-	-
H. Lee et al. [41], 2019	Sleep-Stage Classification	OMP	Random binary matrix	-
N. Mammone et al. [36], 2019	Alzheimer’s disease analysis	BSBL	Sparse binary matrix	DCT transform
R. Shrivastwa et al. [40], 2020	Motor imagery	CNN-Based	Bernoulli random matrix	-

Glossary of terms: Alternating Direction Method of Multipliers (ADMM); Basic Pursuit (BP); Basis Pursuit Denoising (BPDN); Block Sparse Bayesian Learning (BSBL); Block Sparse Bayesian Learning-Bounded Optimization (BSBL-BO); Block Sparse Bayesian Learning-Fast Marginalized (BSBL-FM); Compressive Sampling Matching Pursuit (CoSaMP); Graph Fourier Transform and Nonconvex (GFTN); Hard Thresholding Pursuit (HTP); Iteratively Reweighted Least Square (IRLS); Orthogonal Matching Pursuit (OMP); Regularized Cosparsity and Low-Rank (RCS-CLR); Regularized Least-Squares (RLS); Simultaneous Orthogonal Matching Pursuit (SOMP).

**Table 2:** A summary of CS applied EEG studies (signal reconstruction focused).

Studies	Reconstruction Algorithm	Sensing Matrix	Sparse Basis
M. Hosseini et al. [39], 2013	BPDN	Sparse binary matrix	Gabor dictionary
Z. Zhang et al. [51], 2013	BSBL-BO	Sparse binary matrix	DCT dictionary
R. Kus et al. [47], 2013	Multivariate matching pursuit	Random matrix	Gabor dictionary
S. Fauvel et al. [63], 2014	BSBL-BO	Sparse binary matrix	Gabor dictionary
Y. Liu et al. [79], 2015	$l_{0}$ norm and $S_{0}$ norm	2^nd^ order difference matrix	-
B. Kaliannan et al. [38], 2016	DCS-SOMP	Gaussian random matrix	Joint sparsity
H. Mahrous et al. [68], 2016	BSBL-BO	Sparse binary matrix	DCT dictionary
J. Zhu et al. [60], 2016	$l_{q}$ norm and $S_{q}$ norm	Gaussian random matrix	Wavelet dictionary
H. Djelouat et al. [86], 2017	Subspace pursuit	Bernoulli random matrix	Wavelet transform
X. Li et al. [67], 2018	BPDN	Sparse binary matrix	Gabor dictionary
S. Khoshnevis et al. [87], 2019	Kronecker-based technique	Deterministic binary matrix	-
M. Rani et al. [89], 2019	BP, BPDN, OMP, CoSaMP	pseudorandom sequence	Fourier transform
M. Tayyib et al. [80], 2020	ADMM	sparse circulant matrix	Fourier transform
X. Zou et al. [81], 2020	ADMM	2^nd^ order difference matrix	Graph Fourier transform
